# Assessment of Antioxidant and Cytotoxic Activities of Extracts of *Dendrobium crepidatum*

**DOI:** 10.3390/biom9090478

**Published:** 2019-09-12

**Authors:** Mukti Ram Paudel, Mukesh Babu Chand, Basant Pant, Bijaya Pant

**Affiliations:** 1 Central Department of Botany, Tribhuvan University, Kathmandu 44613, Nepal; 2 Annapurna Research Center, Kathmandu 44600, Nepal

**Keywords:** DPPH, extract, GC–MS, MTT, orchid

## Abstract

*Dendrobium crepidatum* is an epiphytic orchid found in south Asia including Nepal and China. This orchid species is widely used in traditional Chinese medicine (TCM) for the treatment of cancer, diabetes, cataracts, and fever. The objectives of the present research were to assess the antioxidant and cytotoxic properties of its stem’s extracts with the identification of bioactive secondary metabolites. The antioxidant and cytotoxic activities were evaluated using the DPPH (2,2-diphenyl-1-picrylhydrazyl) and MTT (3-(4,5-dimethylthiazol-2-yl)-2,5-diphenyltetrazolium bromide) assays, respectively, and compounds were identified using GC–MS (gas chromatography and mass spectrometry). Ethanol and acetone extracts scavenged 94.69 ± 0.10% and 93.41 ± 0.86% of DPPH free radicals, respectively. They showed 50% inhibition of DPPH free radicals (IC_50_) at concentrations of 73.90 µg/mL and 99.44 µg/mL, which were found to be statistically similar to that of ascorbic acid (control). Chloroform extract inhibited the growth of 81.49 ± 0.43% of HeLa (human cervical carcinoma) cells and hexane extract inhibited the growth of 76.45 ± 4.26% of U251 (human glioblastoma) cells at 800 µg/mL concentration. These extracts showed 50% inhibition of cell growth (IC_50_) toward both the HeLa and U251 cell lines at their high concentrations, which were found statistically significantly different from that of cisplatin drug (control). The above extracts showed antioxidant and cytotoxic properties, potentially due to the presence of tetracosane, triacontane, stigmasterol, and some phenol derivatives (2-methoxy-4-vinylphenol, 2-methoxy-5-(1-propenyl)-phenol, *p*-mesyloxyphenol, and 2,6-dimethoxy-4-(2-propenyl)-phenol). This study explores the potential of this orchid in alternative medicine toward the development of drugs from its medicinally active compounds.

## 1. Introduction

*Dendrobium crepidatum* is an epiphytic orchid dispersed in south Asia including Nepal and China [1]. The stems of this orchid are widely used in traditional Chinese medicine (TCM) for the treatment of cancer, diabetes, cataracts, and fever [2,3]. Previous research mainly focused on the isolation of alkaloids and bibenzyl derivatives [4,5,6], the anti-inflammatory properties of homocrepidine [7], and enhancement of neurite outgrowth in PC-12 cells by crepidatuol, confusarin, and 3-(2-acetoxy-5-methoxy)-phenylpropanol [8]. Furthermore, there are no research reports on the biological activities of this orchid species. It is well known that polyphenol compounds are linked with declining the risk of emergent chronic diseases such as cancer, aging, and cardiovascular diseases [9,10]. Natural antioxidant-enriched compounds showed robust defense mechanisms in contrast to the cellular damage caused by free-radical-induced oxidative stress [11,12,13]. Antioxidant-enriched bioactive compounds of plants are particularly useful for precluding cancer by inducing the apoptosis of cancer cells [14,15,16]. Thus, it is significant and obligatory to quantitatively and qualitatively analyze polyphenol compounds and their antioxidant and cytotoxic properties. The present study mainly focuses on the antioxidant and cytotoxic properties of extracts of *Dendrobium crepidatum*.

## 2. Results and Discussion

### 2.1. Total Polyphenols Content

Total phenolic content (TPC) and total flavonoid content (TFC) in the extracts of *Dendrobium crepidatum* (DC) were found to vary (Table 1). The highest amount of TPC (78.11 ± 0.72 µg gallic acid equivalent (GAE)/mg extract) was found in the ethanol extract (DCE). It was found to be significantly different from that of other extracts (*p* ≤ 0.05). Similarly, the highest amount of TFC (82.62 ± 1.13 µg quercetin (QE)/mg extract) was found in the hexane extract (DCH), and the second highest amount of TFC was found in the acetone extract (DCA). They were also found to be significantly different from that of other extracts (*p* ≤ 0.05).

Polyphenols, including phenolic and flavonoid compounds, occur widely in food, originating from plants in a diverse manner. All of them played a vital role in the successful treatment of a wide variety of diseases since ancient times [10,17]. Phenolics and flavonoids were shown to be highly effective scavengers of most oxidizing free radicals [18,19]. Recently, polyphenol compounds gained interest because they exhibit beneficial health effects due to their potential antibacterial, antiviral, anti-inflammatory, and anticancer activities [20,21,22,23]. Polyphenol compounds were found to vary in different solvents because they depend on the polarity of the solvents. The ethanol and acetone extracts exhibited high TPC (78.11 and 61.27 µg GAE/mg extract, respectively), and the hexane, acetone, and ethanol extracts exhibited high TFC (82.62, 71.93, and 61.57 µg QE/mg extract, respectively), similar to previous studies [24,25]. The above results suggest that phenolics and flavonoids are major contributors to the antioxidant and cytotoxic activity.

### 2.2. DPPH Free-Radical Scavenging Potential and Antioxidant Capacity

The scavenging ability of DPPH (2,2-diphenyl-1-picrylhydrazyl) free radicals is widely used to analyze the antioxidant potential. The DPPH free-radical scavenging potential of extracts of *Dendrobium crepidatum* is shown in Table 2. All extracts showed scavenging capacity against the DPPH free radicals. The scavenging percentage of DPPH free radicals varied from 20.13 ± 4.65% for the chloroform extract (DCC) at 50 µg/mL to 94.69 ± 0.10% for the ethanol extract (DCE) at 800 µg/mL. DCE and DCA had the highest and most significant (*p* ≤ 0.05) scavenging percentage of DPPH free radicals at the different concentrations (except at 50 and 100 µg/mL for DCA) as compared to the other extracts. Therefore, DCA and DCE showed the highest antioxidant capacity (lowest IC_50_: 99.44 µg/mL and 73.90 µg/mL, respectively), statistically similar to that of standard ascorbic acid (AA) (IC_50_: 38.21 µg/mL) (Table 3).

The antioxidant activity has a strong relationship with the polyphenol compounds [26,27]. Phenol derivatives present in plants were reported to have multiple biological effects, including antioxidant activity [24,28,29,30,31]. In our experiment, all the extracts of *D. crepidatum* were capable of decolorizing DPPH. The hexane, acetone, ethanol, and methanol extracts exhibited a high degree of DPPH free-radical scavenging activity (88.17%, 93.41%, 94.69%, and 85.23%, respectively, at 800 µg/mL) because they had a high content of TPC and TFC [24,32,33]. However, in the statistical comparison of the IC_50_ value of extracts with ascorbic acid, only acetone and ethanol extracts showed similarity with the IC_50_ of ascorbic acid. The free-radical scavenging activities of these extracts depend on the ability of their antioxidant-enriched compounds to lose hydrogen. DPPH free radicals can easily receive an electron of hydrogen from antioxidant-enriched extracts to become a stable diamagnetic molecule. Natural antioxidant-enriched compounds present in the plants are responsible for inhibiting the deleterious consequences of oxidative stress [34]. When DPPH accepts an electron donated by an antioxidant compound, the DPPH is decolorized and can quantitatively be measured from changes in absorbance [35,36]. Various reports suggested that ascorbic acid, tocopherol, flavonoids, polyphenols, and tannins reduced and decolorize DPPH via their hydrogen-donating ability [37,38,39].

### 2.3. Cytotoxic Activity toward HeLa and U251 Cell Lines

Cytotoxic activity toward the HeLa (human cervical carcinoma) and U251 (human glioblastoma) cell lines was determined. The growth inhibition percentage of HeLa cells by the extracts is shown in Table 4. The growth inhibition percentage of HeLa cells was found to vary from 19.84 ± 4.31% for the hexane extract (DCH) at 100 µg/mL to 81.49 ± 0.43% for the chloroform extract (DCC) at 800 µg/mL. DCC showed a significantly high percentage of growth inhibition of HeLa cells at 800 µg/mL. However, the acetone extract (DCA) showed a high percentage of growth inhibition of HeLa cells at 400 µg/mL (74.35 ± 0.59%), significantly different from other extracts. Similarly, ethanol (DCE) and methanol (DCM) extracts showed a significantly high percentage of growth inhibition of HeLa cells at 100 µg/mL and 200 µg/mL. However, DCH showed the lowest percentage of growth inhibition of HeLa cells.

The growth inhibition percentage of U251 cells by the extracts is shown in Table 5. The hexane extract (DCH) showed a significantly high percentage of growth inhibition of U251 cells at 76.45 ± 4.26% and 70.79 ± 9.72% for 800 µg/mL and 400 µg/mL concentrations, respectively. However, the methanol extract (DCM) showed a significantly higher percentage of growth inhibition of U251 cells growth at 100 µg/mL (21.69 ± 1.22%) and 200 µg/mL (44.43 ± 2.99%) than that of other extracts. 

The cytotoxic capacity (IC_50_) of all the extracts was found to be significantly higher than that of the cisplatin drug (CDDP) (IC_50_: 25.00 ± 0.5 µg/mL) toward both HeLa and U251 cell lines (Table 6).

Many chemotherapeutic drugs and folk medicinal plants exert their anticancer effect by inducing cell apoptosis [40,41,42]. The crude extracts were used to treat adenocarcinoma and glioblastoma cells, showing morphological changes such as a reduction in size and cell volume, cell shrinkage, membrane blebbing, chromatin condensation, nuclear fragmentation, and formation of apoptotic bodies, indicating that the extracts induced apoptosis [43]. The hexane extract showed growth inhibition of 76.45% of U251 cells at 800 µg/mL concentration. The reason behind this is that some anticancer compounds such as tetracosane and triacontane were identified in the hexane extract (Table 7), which play a significant role in cytotoxic activity. Similarly, the chloroform extract inhibited the growth of 81.49% of HeLa cells and 71.24% of U251 cells at 800 µg/mL concentration. Many phenolic and flavonoid derivatives present in the chloroform extract were identified, which showed cytotoxic activity (Table 7). However, considering the cytotoxic capacity (IC_50_) values, the methanol extract had better cytotoxic capacity toward both HeLa and U251 cell lines because this extract had many polyphenol derivatives (Table 7) that play a vital role in the inhibition of growth of cancer cells. However, the cytotoxic capacity of all extracts was found to be significantly different from that of the commercial anticancer drug (cisplatin). The result clearly indicated that the extracts have some cytotoxic compounds, but other compounds are also present that may attribute to the cytotoxic capacity of the extracts as compared to the cisplatin. Previous reports enlightened that the methanol extract of some *Dendrobium* species showed cytotoxicity toward the HeLa and U251 cell lines [32,33,44]. Our results support the previous studies, which showed that polyphenol derivatives are responsible for the induction of apoptosis in cancer cells [45].

### 2.4. Detection and Identification of Compounds by GC–MS

The GC–MS (gas chromatography and mass spectrometry) analysis showed the presence of a wide variety of compounds in the extracts. Detected ions were identified via the use of a standard molecular formula. The detected and identified compounds in the extracts with peak area percentage are listed in Table 7.

9,12-Octadecadienoic acid, methyl ester (17.27%) was found as a high constituent in the DCH. Furthermore, many alkaloids such as crepidamine (3.79%), isocrepidamine (2.80%), crepidine (3.19%), heneicosane (1.25%), tetracosane (3.74%), and triacontane (2.65%) were present. Some phenol derivatives such as 4-methyl, 1-naphthalenol (0.40%), 1-heptacosanol (1.44%), α-tocopherol (2.66%), and stigmasterol (2.03%) were also detected. DCC had many active compounds of alkaloids, phenols, and ester derivatives. Some identified phenol derivatives were 1-heptacosanol (0.75%), 1-eicosanol, trifluoroacetate (0.70%), 5-sec-butylpyrogallol (5.54%), 1-nonadecanol (1.10%), indolizinol (5.23%), 1-heneicosanol (0.69%), terpineol (0.86%), and naphthol (0.97%). One amino-acid (tryptophan) derivative (2.21%) was also identified. Similarly, DCA also had many important secondary metabolites like alkaloids, phenols, and esteroids. The major compounds present in DCA were dimethylsulfoxonium formylmethylide (7.15%), 2-phenylethanol (0.94%), l-α-terpineol (1.12%), 2-methoxy-4-vinylphenol (0.84%), 4-methyl-1-naphthol (1.20%), eugenol (1.05%), isocrepidamine (2.28%), crepidine (7.08%), crepidamine (10.17%), tryptophan (1.14%), gephyrotoxin 207a (0.64%), 2-hydrazino-6-methylpyrimidin-4-ol (7.01%), 1-(2-hydroxy-3,4-dimethoxyphenyl)-2-(4-methoxyphenyl) ethanone (5.20%), and 2-(1-methylimidazol-4-yl)-1,2,3,4-tetrahydroquinoline (7.44%). DCE also had alkaloids and phenol derivatives. Some phenol derivatives were 2-methoxy-4-vinylphenol (2.22%), 4-methyl-1-naphthalenol (0.99%), 2-methoxy-5-(1-propenyl)-phenol (0.79%), *p*-mesyloxyphenol (0.49%), 2,6-dimethoxy-4-(2-propenyl)-phenol (1.08%), and 7-phenyl-1,5-dihyro-imidazo [4,5-d] pyridazin-4-one (1.64%). Some active alkaloids were crepidamine (4.50%), crepidine (0.37%), quinoline (2.70%), diethyl phthalate (0.80%), and tridemorph (3.08%). Some of the compounds identified in DCM were dimethylsulfoxonium formylmethylide (14.94%), disiloxane (15.96%), dibutyl phthalate (2.79%), crepidamine (1.43%), and crepidine (0.56%).

Some of the constituents revealed by GC–MS are biologically active compounds. Dimethylsulfoxonium formylmethylide leads to antimicrobial properties. Tetracosane showed cytotoxic activity against cancer cell lines [46]. Triacontane possesses antibacterial, antidiabetic, and antitumor activities [3]. Tetradecanoic acid and hexadecanoic acid have antioxidant and antimicrobial activities [47]. Many phenol derivatives present in the extracts were also proven to have antioxidant and anticancer activities [2].

## 3. Conclusions

It can be concluded that extracts of *D. crepidatum* show antioxidant and cytotoxic activities. The extracts have many bioactive compounds such as tetracosane, triacontane, tetradecanoic acid, hexadecanoic acid, and phenol derivatives, leading to antioxidant and cytotoxic activities. Hence, this orchid species might be used in alternative medicine to develop pharmaceutical drugs from isolated bioactive compounds.

## 4. Materials and Methods

### 4.1. Plant Materials

Stems of *Dendrobium crepidatum* were collected during March and April from Makawanpur district (27°33’44” north (N), 85°00’45” east (E)) of central Nepal. The voucher specimen (M05) was deposited in the Tribhuvan University Central Herbarium (TUCH), Kathmandu, Nepal. The stems were air-dried at room temperature.

### 4.2. Preparation of Extracts

The powdered form of air-dried stems was extracted with hexane, chloroform, acetone, ethanol, and methanol in the ratio of 1:10 of the weight of powder and volume of solvent (*w*/*v*) using the Soxhlet extraction method. About 30 g of powdered stem was extracted with 300 mL of each solvent. The solvent was evaporated at room temperature, and extracts dissolved in ethanol were kept at 4 °C for further experiments.

### 4.3. Quantification of Total Polyphenol Content

#### 4.3.1. Quantification of Total Phenolic Content

Total phenolic content (TPC) was quantified by a spectrophotometric method using Folin–Ciocalteu (FC) reagent [48]. For a test sample, 0.5 mL of extract (1 mg/mL stock concentration in ethanol) was consistently mixed with 2.5 mL of 10% FC reagent and 2.5 mL of 7.5% sodium bicarbonate. For a blank sample, 0.5 mL of ethanol was mixed with 2.5 mL of 10% FC reagent and 2.5 mL of 7.5% sodium bicarbonate. Both the reaction mixtures were incubated at room temperature for 45 min and their absorbance was measured using a Genesys ultraviolet (UV)–visible light spectrophotometer (Thermo Fisher Scientific, Waltham, MA, USA) at 765 nm. TPC was expressed in micrograms of gallic acid equivalent (GAE) per milligram of dry extract using the following equation:

(1) $$C=\frac{c\times V}{m}$$

where *C* is the total phenolic content (μg/mg extract in GAE), *c* is the concentration of gallic acid established from the calibration curve (μg/mL), *V* is the volume of extract in mL, and *m* is the weight of plant extract in mg.

**Figure 1:**
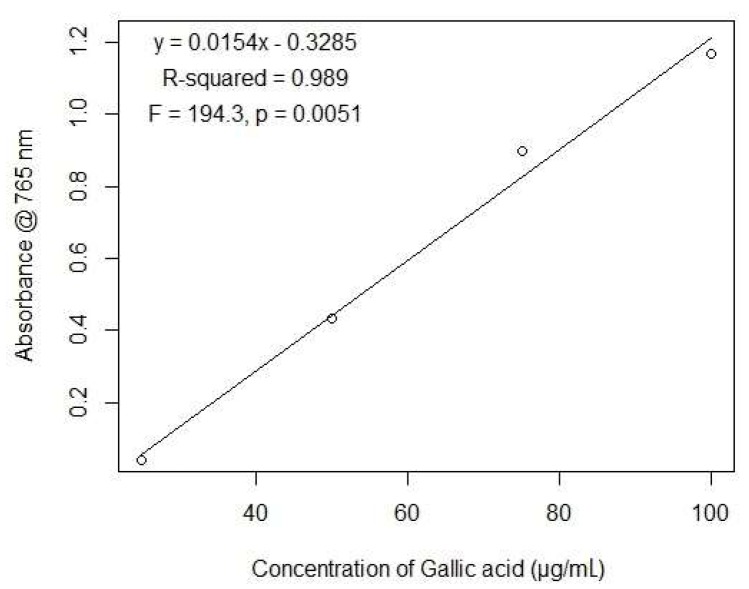
Calibration curve of gallic acid obtained from the linear model.

TPC was calculated using the equation of gallic acid obtained from the linear regression model, where the abscissa represents the concentration of gallic acid, and the ordinate represents the triplicate of absorbance at each concentration (Figure 1).

#### 4.3.2. Quantification of Total Flavonoid Content

Total flavonoid content (TFC) was quantified by a spectrophotometric method using aluminum chloride [48]. For a test sample, 1 mL of extract (1 mg/mL stock concentration in ethanol) was mixed with 1 mL of 2% aluminum chloride. For a blank sample, 1 mL of ethanol was mixed with 1 mL of 2% aluminum chloride. Both the reaction mixtures were incubated for an hour at room temperature, and their absorbance was measured using a Genesys UV–visible light spectrophotometer (Thermo Fisher Scientific) at 415 nm. TFC was expressed in micrograms of quercetin equivalent (QE) per milligram of dry extract using the following equation:

(2) $$C=\frac{c\times \;V}{m}$$

where *C* is the total flavonoid content (μg/mg extract in QE), *c* is the concentration of quercetin established from the calibration curve (μg/mL), *V* is the volume of extract in mL, and *m* is the weight of plant extract in mg.

**Figure 2:**
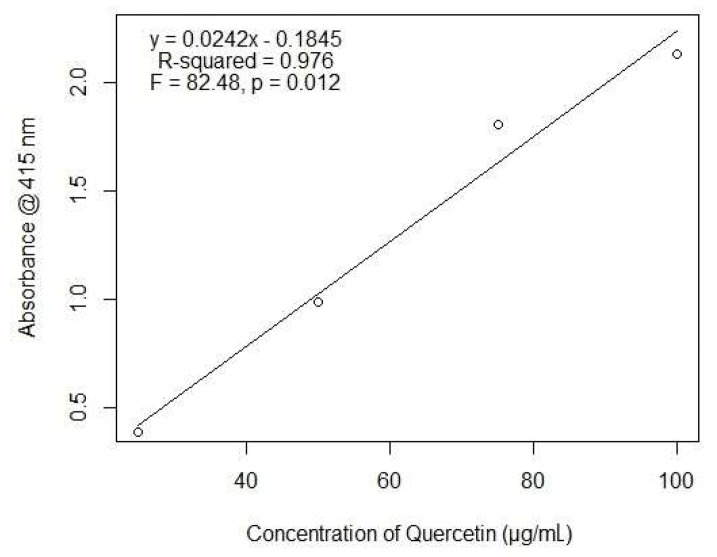
Calibration curve of quercetin obtained from the linear model.

TFC was calculated using the equation of quercetin obtained from the linear regression model, where the abscissa represents the concentration of quercetin, and the ordinate represents the triplicate of absorbance at each concentration (Figure 2).

### 4.4. Evaluation of Antioxidant Properties

The antioxidant activity was determined using a DPPH (2,2-diphenyl-1-picrylhydrazyl) free-radical scavenging assay [36,49]. The extract (1 mg/mL concentration in ethanol) was prepared in a series of concentrations (50, 100, 200, 400, and 800 μg/mL). About 1.5 mL of extract of various concentrations was mixed with 1.5 mL of ethanolic DPPH solution (0.25 mM). The reaction mixture was shaken vigorously and kept for 30 min to reach a steady state at room temperature. The change of purple 2,2-diphenyl-1-picrylhydrazyl to yellow 2,2-diphenyl-1-picrylhydrazine due to the action of secondary metabolites of the extract was determined by measuring the absorbance at 517 nm using a Genesys UV–visible light spectrophotometer (Thermo Fisher Scientific). Ascorbic acid was used as the positive control in which the abovementioned procedure was followed. The percentage of DPPH free-radical scavenging activity was calculated using the formula given below.

(3) $$Percentage\;of\;DPPH\;scavenge\;rate=1-\frac{A1-A2}{A0}\times 100,$$

where *A*0 is the mean absorbance of the control (only DPPH, without extract), *A*1 is the mean absorbance of the extract with DPPH, and *A*2 is the mean absorbance of the extract without DPPH.

Antioxidant capacity was expressed as IC_50_ (concentration required for 50% inhibition of DPPH free radicals). The IC_50_ value was calculated using a linear, second- or third-order polynomial regression equation obtained from the model, where the abscissa represents the series of concentrations, and the ordinate represents the percentage of DPPH free-radical scavenging activity.

### 4.5. Evaluation of Cytotoxic Properties

#### 4.5.1. Human Cancer Cell Culture

Human brain glioblastoma cells (U251) were obtained from the Department of Neurosurgery of Kagoshima University, Japan, and human cervix adenocarcinoma cells (HeLa) were obtained from Shikhar Biotech Pvt. Ltd., Nepal. These cells were cultured in a T-flask containing Roswell Park Memorial Institute (RPMI-1640) medium supplemented with l-glutamine, penicillin/streptomycin, and 10% fetal bovine serum (FBS) at 37 °C and 5% CO_2_. Once cells obtained more than 80% confluence, cells were again sub-cultured in the new medium after trypsinization.

#### 4.5.2. MTT Assay

Cytotoxicity was determined using the MTT colorimetric assay [50,51]. From the cell suspension, 5 × 10^3^ cells in 100 μL of medium were dispensed into a 96-well microtiter cell culture plate and incubated under the abovementioned conditions of cell culture for 48 h. About 100 μL of extract (ethanolic extract diluted in medium) of four cytotoxic concentrations (100, 200, 400, and 800 μg/mL) was added into the well of the cell culture plate and then re-incubated for 24 h. Then, 10 µL of MTT (5 mg/mL) was added to the well, and the cell plate was re-incubated for another 4 h. The live cells were converted into purple insoluble formazan crystals due to the oxidoreductase enzyme secreted by mitochondria. The formazan crystals of living cells were dissolved in 100 μL of dimethyl sulfoxide (DMSO), and then the plate was read in a microplate reader (iMark^TM^, Bio-Rad, Hercules, CA, USA) at 595 nm. Cisplatin drug (CDDP) was used as the positive control in which the abovementioned procedure was followed. The percentage of cell growth inhibition was calculated using the following formula:

(4) $$Percentage\;of\;cells\;growth\;inhibition=100-\frac{A\left(cs\right)-A\left(m\right)}{A\left(cc\right)-A\left(m\right)}\times 100\%,$$

where *A(cs)* is mean absorbance of treated cells with test samples, *A(cc)* is mean absorbance of untreated cells, and *A(m)* is mean absorbance without cells.

A dose–response curve was plotted to calculate the 50% inhibition of cell growth (IC_50_). The cytotoxic capacity (IC_50_) was calculated using a linear, second- or third-order polynomial regression equation, where the abscissa represents the series of concentrations, and the ordinate represents the triplicate percentage of inhibition of cell growth.

### 4.6. Identification of Compounds by GC–MS

Secondary metabolites were detected and identified using GC–MS (Shimadzu Europa GmbH, Duisburg, Germany). Spectroscopic detection involved an electron ionization system which utilized high-energy electrons (70 eV). Pure helium gas (99.99%) was used as the carrier gas with a column flow rate of 0.95 mL/min. The initial temperature was set to 100 °C with an increasing rate of 3 °C/min and a holding time of about 10 min. Finally, the temperature was increased to 300 °C at 10 °C/min. One microliter of the prepared 1% extract was injected in splitless mode. The relative quantity of the chemical compounds present in each extract was expressed as a percentage based on the peak area produced in the chromatogram. Bioactive compounds were identified based on GC retention time and matching of the spectra with data of standards.

### 4.7. Statistical Analysis

All the data were presented as means and standard deviations of three analyses. Duncan’s test was applied to compare the mean differences among the five extracts for total polyphenol content, percentage of DPPH free-radical scavenging activity, and percentage inhibition of HeLa and U251 cell growth. Dunnett’s test was applied to compare the mean differences between the extracts and positive control for IC_50_ values for determination of antioxidant and cytotoxic capacity.

## Figures and Tables

**Table 1:** Total phenolic and flavonoid contents in the extracts.

Extract	TPC (µg GAE/mg Extract)	TFC (µg QE/mg Extract)
**DCH**	45.34 ± 5.60^c^	82.62 ± 1.13^a^
**DCC**	37.18 ± 0.70^d^	53.36 ± 0.83^d^
**DCA**	61.27 ± 3.55^b^	71.93 ± 1.59^b^
**DCE**	78.11 ± 0.72^a^	61.57 ± 3.46^c^
**DCM**	28.89 ± 2.11^e^	40.14 ± 0.25^e^

The values are expressed as means ± standard deviation; values in a column with different superscript letters are statistically significantly different at *p* ≤ 0.05. DCH: *Dendrobium crepidatum* hexane; DCC: *D. crepidatum* chloroform; DCA: *D. crepidatum* acetone; DCE: *D. crepidatum* ethanol; DCM: *D. crepidatum* methanol; TPC: total phenolic content; TFC: total flavonoid content; GAE: gallic acid equivalent; QE: quercetin equivalent.

**Table 2:** Percentage of 2,2-diphenyl-1-picrylhydrazyl (DPPH) free radicals scavenged by the extracts at different concentrations.

Plant Extract	Concentration of Extract (µg/mL)				
	50	100	200	400	800
**DCH**	35.14 ± 2.89^b^	41.43 ± 5.08^b^	50.31 ± 6.20^b^	60.01 ± 13.55^b^	88.17 ± 5.60^a^
**DCC**	20.13 ± 4.65^c^	23.43 ± 6.22^c^	42.71 ± 3.98^c^	59.36 ± 3.53^b^	74.11 ± 11.34^b^
**DCA**	34.54 ± 4.62^b^	46.31 ± 1.74^b^	78.53 ± 0.59^a^	92.76 ± 1.86^a^	93.41 ± 0.86^a^
**DCE**	44.02 ± 7.22^a^	64.31 ± 0.62^a^	84.36 ± 2.47^a^	94.10 ± 0.26^a^	94.69 ± 0.10^a^
**DCM**	40.03 ± 1.24^ab^	43.90 ± 2.46^b^	55.42 ± 2.43^b^	69.92 ± 0.22^b^	85.23 ± 5.57^ab^

The values are expressed as means ± standard deviation; values in a column of concentration with the same superscript letter are not statistically significantly different at *p* ≤ 0.05. DCH: *D. crepidatum* hexane; DCC: *D. crepidatum* chloroform; DCA: *D. crepidatum* acetone; DCE: *D. crepidatum* ethanol; DCM: *D. crepidatum* methanol.

**Table 3:** Half maximal inhibitory concentration (IC_50_; µg/mL) of the plant extracts and ascorbic acid (AA) (control) toward DPPH free-radical scavenging activity.

Plant Extracts and Ascorbic Acid (AA)						
	DCH	DCC	DCA	DCE	DCM	AA
**IC_50_**	306.77 ± 51.14*	277.08 ± 27.80*	99.35 ± 2.40	74.00 ± 5.75	186.39 ± 72.79*	38.21 ± 2.00

The values are expressed as means ± standard deviation; values with an asterisk are statistically significantly different from ascorbic acid at *p* ≤ 0.05. DCH: *D. crepidatum* hexane; DCC: *D. crepidatum* chloroform; DCA: *D. crepidatum* acetone; DCE: *D. crepidatum* ethanol; DCM: *D. crepidatum* methanol; AA: ascorbic acid.

**Table 4:** Percentage of HeLa cell growth inhibition by the extracts at different concentrations.

Plant Extract	Concentration of Extract (µg/mL)			
	100	200	400	800
**DCH**	19.84 ± 4.31^c^	31.96 ± 3.57^c^	58.16 ± 0.71^c^	62.10 ± 5.04^c^
**DCC**	20.96 ± 1.67^c^	24.56 ± 1.17^d^	55.25 ± 3.95^c^	81.49 ± 0.43^a^
**DCA**	25.97 ± 1.90^bc^	42.06 ± 2.28^b^	74.35 ± 0.59^a^	78.21 ± 2.53^ab^
**DCE**	30.05 ± 1.87^ab^	48.66 ± 0.10^a^	52.89 ± 1.48^c^	59.55 ± 0.63^c^
**DCM**	35.61 ± 3.92^a^	47.40 ± 1.24^a^	67.01 ± 1.97^b^	73.05 ± 1.39^b^

The values are expressed as means ± standard deviation; values in a column of concentration with the same superscript letter are not statistically significantly different at *p* ≤ 0.05. DCH: *D. crepidatum* hexane; DCC: *D. crepidatum* chloroform; DCA: *D. crepidatum* acetone; DCE: *D. crepidatum* ethanol; DCM: *D. crepidatum* methanol.

**Table 5:** Percentage of U251 cell growth inhibition by the extracts at different concentrations.

Plant Extract	Concentration of Extract (µg/mL)			
	100	200	400	800
**DCH**	16.35 ± 4.26^b^	18.26 ± 0.83^b^	70.79 ± 9.72^a^	76.45 ± 4.26^a^
**DCC**	9.76 ± 2.71^c^	23.02 ± 11.33^b^	39.96 ± 8.25^c^	71.24 ± 10.98^a^
**DCA**	20.82 ± 4.14^a^	16.94 ± 1.65^b^	41.06 ± 14.03^c^	52.53 ± 6.60^c^
**DCE**	15.31 ± 5.73^b^	26.89 ± 6.48^b^	49.62 ± 23.55^b^	61.28 ± 11.96^b^
**DCM**	21.69 ± 1.22^a^	44.43 ± 2.99^a^	54.36 ± 0.21^b^	64.41 ± 6.49^b^

The values are expressed as means ± standard deviation; values in a column of concentration with the same superscript letter are not statistically significantly different at *p* ≤ 0.05. DCH: *D. crepidatum* hexane; DCC: *D. crepidatum* chloroform; DCA: *D. crepidatum* acetone; DCE: *D. crepidatum* ethanol; DCM: *D. crepidatum* methanol.

**Table 6:** IC_50_ (µg/mL) of the plant extracts and cisplatin (CDDP) (control) toward the HeLa and U251 cell lines.

Plant Extracts and Cisplatin (CDDP)						
	DCH	DCC	DCA	DCE	DCM	CDDP
**IC_50_ HeLa**	325.38 ± 5.03*	369.16 ± 3.03*	229.27 ± 3.80*	223.07 ± 2.86*	194.14 ± 4.94*	25.00 ± 0.5
**IC_50_ U251**	314.70 ± 4.20*	539.58 ± 1.04*	687.34 ± 6.83*	558.63 ± 3.61*	301.99 ± 2.00*	25.00 ± 0.5

The values are expressed as means ± standard deviation; values with an asterisk are statistically significantly different from CDDP at *p* ≤ 0.05. DCH: *D. crepidatum* hexane; DCC: *D. crepidatum* chloroform; DCA: *D. crepidatum* acetone; DCE: *D. crepidatum* ethanol; DCM: *D. crepidatum* methanol; CDDP: cisplatin drug).

**Table 7:** Identified compounds from the extracts of *D. crepidatum* by GC–MS. RT—retention time.

RT (min)	Compound Name	Peak Area Percentage in the Extract				
		DCH	DCC	DCA	DCE	DCM
4.060	Dimethylsulfoxonium formylmethylide			7.15		14.94
4.253	2-Phenylethanol			0.94		
4.261	1-Butoxy-2-propanol acetate				4.14	
4.340	2,3-dihydro-3,5-dihydroxy-6-methyl-4*H*-Pyran-4-one				5.21	13.60
4.375	2,5-Dimethyl-4-hydroxy-3(2*H*)-furanone				4.56	
4.585	(l)-alpha-Terpineol			1.12		
4.652	(−)-alpha-Terpineol		0.86			
4.806	2-Furaldehyde			5.78		
5.217	5-(Hydroxymethyl)- 2-Furancarboxaldehyde				21.90	1.39
5.282	2-Methoxy-4-vinylphenol			0.84	2.22	
5.430	4-methyl-1-naphthalenol	0.40			0.99	
5.458	4-Methyl-1-naphthol		0.97	1.20		
5.528	Eugenol			1.05		
5.672	2-Methoxy-5-(1-propenyl)- phenol				0.79	
6.286	*N*,*N*’-Dibutylidene-hydrazine				0.46	
6.768	*p*-Mesyloxyphenol				0.49	
7.285	, 2,6-Dimethoxy-4-(2-propenyl)-phenol				1.08	
7.362	1,7-Trimethylene-2,3,5-trimethylindole	3.93				
7.370	1-(3,4-Dihydro-1-naphthalenyl)- Pyrrolidine		4.22	1.04		
7.495	3-(2-Phenylethylamino)-5-(4-methylphenyl)-2-cyclohexen-1-one				1.25	
7.740	2-Benzylhexahydropyrrolizin-3-one			1.03	1.07	
8.830	5-Acetyl-4-amino-3-(2-*N*-pyrrolidinylethylthio) thieno [3,2-d]isothiazole				2.58	
8.841	2-*t*-Butyl-4-quinolinealdehyde			6.54		
8.851	2,3-Dihydro-2,4,8-trimethyl-furo [2,3-b] quinoline	5.82				0.99
8.861	1,3,4,10-Tetrahydro-2-methyl-9(2*H*)-Acridinone		5.96			
9.097	1,2-Benzenedicarboxylic acid, bis (2-methylpropyl) ester	2.05	1.38		1.19	16.08
9.099	Diisobutyl phthalate			3.73		
9.547	4-Acetyl-3-amino-5-butyl-2,4-cyclopentadiene-1,1,2-tricarbonitrile	2.09	3.40			
9.551	*N*-(*N*’-Ethoxycarbonylisonipecotinoyl)-isonipecotic acid ethyl ester			3.78		
9.611	2,3,9-Trimethyl-5-trimethylsilyloxy-2-azabicyclodecane				1.44	
9.680	2-Butoxy-5-(4-methylphenyl)- benzaldehyde	2.26				
9.687	5,7-Dimethyl-9-hydroxy-2,3-dihydro-1*H*-cyclopenta [b] quinoline		3.45	4.30		
9.724	7-Phenyl-1,5-dihydro-imidazo [4,5-d] pyridazin-4-one				1.64	
9.733	Dibutyl phthalate					2.79
9.801	1-Heneicosanol		0.69			
10.837	2,4-Dimethyl-3a-phenyldecahydrofuro[3,2-E] indolizin2-ol		5.23			
11.191	1-Nonadecanol		1.10			
11.415	9,12-Octadecadienoic acid, methyl ester	17.27				
11.574	6-Isopentyl-2-*tert*-pentyl-tryptophan methyl ester			1.14		
11.592	2-(1,1-Dimethylpropyl)-6-(3-methylbutyl)-tryptophan methyl ester		2.21		0.32	
11.899	2-Dodecyl-5-methylpyrrolidine		1.60			
11.997	Octahydro-2*H*-Inden-2-one, *cis*-oxime			1.44		
12.175	5-sec-butylpyrogallol		5.54			
12.187	1,2-Dihydro-2-(2-oxocyclohexylidene) quinoline			5.02		
12.269	Isocrepidamine	2.80		2.28		
12.326	1-(6-Hydroxy-7-methyl-6-phenyloctahydro-5-indolizinyl) acetone		3.73			
12.438	1-Eicosanol, trifluoroacetate		0.70			
12.728	2-(Diethylamino)-7-methyl-7phenyl-2,4-cycloheptadien-1-one		2.26			
12.802	2,4-Cycloheptadien-1-one, 2-(diethylamino)-7-methyl-7-phenyl	2.37			0.36	
12.915	3-Bornanone, oxime				1.89	
12.931	3,12-Didehydro-9,10-dimethoxy-galanthan-1,2-diol		4.66			
13.064	iso-Propyl 9-*cis*,11-*trans*-octadecadienoate	5.48				
13.092	4-Methoxy-6-(1-pyrrolidinylmethyl)-1,3,5-triazin-2-amine		6.48			
13.093	2-Hydrazino-6-methylpyrimidin-4-ol			7.01		
13.125	Tetracosane	3.74				
13.135	Crepidamine	3.79		10.17	4.50	1.43
13.139	9-Methoxy-9-Borabicyclo [3.3.1] nonane					1.32
13.190	Gephyrotoxin 207a		3.51	0.64		
13.253	2-Methoxy-3-(1-methylpropyl)- pyrazine	3.14				
13.399	1-(2-Hydroxy-3,4-dimethoxyphenyl)-2-(4-methoxyphenyl) ethanone			5.20		
13.410	1-[2-(5-Hydroxy-1,1-dimethylhexyl)-3-methyl-2-cyclopropen-1-yl]-ethanone		2.93			
13.464	*N*-(9*H*-Xanthen-9-yl)- 4-pyridinecarbothioamide		3.25			
13.519	2-Hydroxy-3,4-dimethoxy-alpha-(*p*-methoxyphenyl) acetophenone	4.15			0.54	
13.537	(−)-Nortrachelogenin		2.60			
13.793	Heneicosane	1.25				
13.942	Crepidine	3.19	4.87	7.08	0.37	0.56
14.041	Dimethoxymethylphenylsilane		3.30	1.75		
14.544	*n*-Tetratriacontane		3.42			
14.599	Tetratriacontane	8.10				
14.767	Monolinolein		0.67			
14.791	*n*-Propyl 9,12-octadecadienoate	3.01				
14.913	*cis*-3,3,5-Trimethyl-cyclohexanol acetate	0.87				
15.019	6-(6-Acetonyl-5-hydroxy-1,4-dimethyl-5-phenyl-2-piperidyl)-3-hexen-2-one		1.34			
15.240	Hexatriacontane	0.90				
15.796	Oxalic acid, di-(1-menthyl) ester	2.07				
15.945	*n*-Hexatriacontane		1.22			
15.960	Triacontane	2.65				
16.050	1-Heptacosanol	1.44	0.75			
17.611	gamma-Tocopherol	1.37				
18.477	alpha-Tocopherol-beta-d-mannoside	2.19				
19.908	(3beta)-ergost-5-en-3-ol	0.85				
20.355	Stigmasterol	2.03				
21.115	2-(1-methylimidazol-4-yl)-1,2,3,4-tetrahydroquinoline			7.44		
21.159	1,2,3,4-Tetrahydro-2-(1-methylimidazol-4-yl)-quinoline	2.53			2.70	
21.424	Estrone-3-acetate-17-methyloxime		3.98			1.05
23.109	dl-alpha-Tocopherol	2.66				
24.466	3,4,5-Trimethoxy-nitrobenzene	0.60	1.69			2.62
